# Dissecting the determinants of malaria chronicity: why within-host models struggle to reproduce infection dynamics

**DOI:** 10.1098/rsif.2014.1379

**Published:** 2015-03-06

**Authors:** Lauren M. Childs, Caroline O. Buckee

**Affiliations:** 1 Center for Communicable Disease Dynamics, Harvard T. H. Chan School of Public Health, Boston, MA 02115, USA; 2 Department of Epidemiology, Harvard T. H. Chan School of Public Health, Boston, MA 02115, USA

**Keywords:** malaria, chronic infection, within-host dynamics, epidemiological model

## Abstract

The duration of infection is fundamental to the epidemiological behaviour of any infectious disease, but remains one of the most poorly understood aspects of malaria. In endemic areas, the malaria parasite *Plasmodium falciparum* can cause both acute, severe infections and asymptomatic, chronic infections through its interaction with the host immune system. Frequent superinfection and massive parasite genetic diversity make it extremely difficult to accurately measure the distribution of infection lengths, complicating the estimation of basic epidemiological parameters and the prediction of the impact of interventions. Mathematical models have qualitatively reproduced parasite dynamics early during infection, but reproducing long-lived chronic infections remains much more challenging. Here, we construct a model of infection dynamics to examine the consequences of common biological assumptions for the generation of chronicity and the impact of co-infection. We find that although a combination of host and parasite heterogeneities are capable of generating chronic infections, they do so only under restricted parameter choices. Furthermore, under biologically plausible assumptions, co-infection of parasite genotypes can alter the course of infection of both the resident and co-infecting strain in complex non-intuitive ways. We outline the most important puzzles for within-host models of malaria arising from our analysis, and their implications for malaria epidemiology and control.

## 1. Introduction

Each year, nearly 200 million people are infected with the malaria parasite, *Plasmodium falciparum*, although only a fraction of infections result in clinical symptoms such as fever or severe anaemia [1]. One of the most notable features of malaria is the variable course and duration of blood-stage infection experienced by different individuals, ranging from high parasite density episodes causing severe disease to persistent, chronic infections that are often undetectable by the standard method of microscopy [1–3]. In areas where malaria is endemic, older children and adults rarely exhibit severe symptoms, suffering only mild or asymptomatic infections. It is thought that this naturally acquired immunity against clinical disease is generated following repeated exposure to genetically diverse parasites. However, high parasite population diversity, as well as the lack of sensitive genetic markers characterizing different genotypes, makes it difficult to distinguish between co-infection, recrudescence or re-infection of an individual [4–6]. As a result, the measured duration of an individual infection with *P. falciparum* in similar settings is highly variable, and the impact of immunity and co-infection on infection length is essentially unknown. Because asymptomatic infections, which are often long-lasting, are critical to the transmission potential of malaria [7–9], and infection length is a key epidemiological parameter in mathematical models predicting the impact of control programmes, this knowledge gap represents a significant hurdle for the design of control and elimination strategies.

Some of the best data on the dynamics of human malaria infections come from experimental infections in neurosyphilis patients undergoing ‘malaria therapy’ in the first half of the twentieth century. Although these patients—malaria-naive and suffering from tertiary syphilis—are not representative of endemic populations, the detailed records provide important insights into the dynamics of parasite density and the remarkable range of infection lengths in untreated infections, from 8 to 417 days. These data form the basis of assumptions such as average duration of infection in many models of malaria transmission [10–18] including those used to inform control. Field studies examining survival times of parasites in the blood have produced widely varying estimates of infection length using a variety of different genetic and statistical methods [19,20]; however, one recent estimate suggests that very short infections—on the order of days rather than weeks—may be more common than previously thought [21]. Monitoring the duration of low-density chronic infections *in vivo* remains challenging, whereas the lack of sensitive genetic markers makes it difficult to measure the dynamics of individual parasite genotypes in the host, all complicating estimates of infection length in endemic settings.

Co-infection with multiple genotypes, which is common in high endemicity settings, represents an additional challenge to understanding chronicity and the impact of heterogeneous infection lengths on transmission [19]. Little evidence exists as to whether co-infection in the human host increases or decreases infection length or infectivity to the mosquito vector, and many transmission models simply assume either that genotypes circulate completely independently or that one strain succeeds and is the sole contributor to onward transmission [22–27]. In others, co-infection is omitted altogether [28,29]. Because there is ample evidence that in areas of high endemicity the majority of infections contain multiple clones [19], and that the frequency of mixed infections changes in different transmission settings, these assumptions are likely to significantly alter model results at the population level.

In the absence of data on the dynamics of individual infections in an endemic setting, which is extremely difficult to measure directly, mathematical models provide important tools to predict the *in vivo* consequences of molecular and immunological mechanisms elucidated from field and *in vitro* studies. Several mathematical frameworks have been developed to quantify parasite dynamics within the blood stage of *P. falciparum* [12,13,30–40], often focusing on infections in naive patients prior to the development of adaptive immune responses [31,34]. All current models suffer from increased complexity caused by the juxtaposition of the discrete parasite life cycle with egress every 48 h, and the more continuously varying immune cell population [12,13,30,32–35,37,40]. As a result, all models require an extensive number of parameters, few of which can be measured directly from experimental data, and despite highly complex model structures, not only are the dynamics of individual malaria therapy patients hard to reproduce, but also chronicity *per se* is difficult to achieve.

Here, we use a mathematical model to test whether reasonable and frequently made biological assumptions about mechanisms of immunity against *P. falciparum* reliably produce the basic features of infection dynamics observed in untreated patients. Most models are not specifically designed to understand the distribution of infection lengths, and therefore do not examine the consequences of their assumptions beyond the scope of their particular question. We find that dramatic changes in the outcome of infection occur with similar combinations of parameters, even in our deterministic framework. We show that this rugged landscape of model outcomes with similar parameters means that chronicity is not a consistent outcome in the presence of natural variation in hosts and parasites. Thus, for this complex system, the standard sensitivity analyses reported by most within-host modelling studies are insufficient. Furthermore, when chronic infections are likely, we examine the impact of co-infection on infection length, and show that the presence of multiple genotypes may significantly alter the persistence of parasites in the human host in unpredictable ways, favouring either the resident or the co-infecting strain, and occasionally both. Our results suggest that commonly employed representations of the within-host processes underlying malaria transmission greatly impact the reliability of models designed to aid control and elimination strategies.

## 2. Results and discussion

### 2.1. Key characteristics of chronic malaria infections

**Figure 1.**
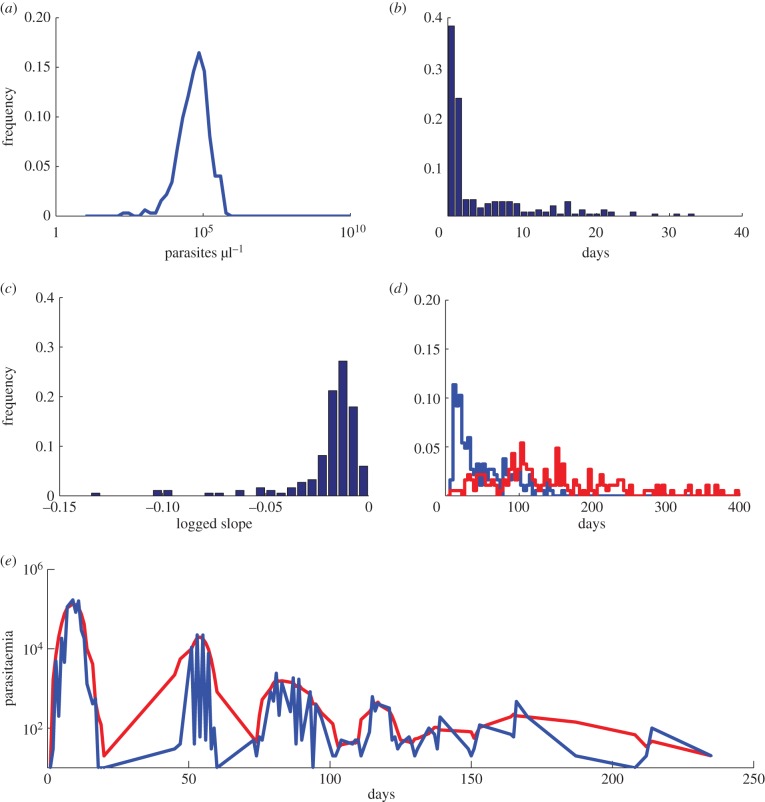
Characteristics of *P. falciparum* dynamics in malaria therapy patients. (*a*) Histogram of the height of the initial peak in parasitaemia of malaria therapy patients. Many patients exhibit initial peak parasitaemia near 10^5^ parasites per microlitre. (*b*) Histogram of the length of subpatent parasitaemia following the first peak in parasitaemia. More than 60% of untreated malaria therapy patients experience subpatent parasitaemia shortly following the initial parasitaemia peak, but the length of time subpatent ranges widely. (*c*) Histogram of the slope from the height of the initial peak in parasitaemia to the final patent parasitaemia on a log scale. Most patients show a similar logged slope that decays slowly. (*d*) Histogram of the day patients are first subpatent (blue) and the day they are last patent (red). Most patients have parasitaemia that drops below detectability long before the infection disappears. (*e*) Individual patient parasitaemia (representative patient shown by the blue line) exhibit short-time oscillations every 48 h and long-time ones on the order of weeks. The red line represents smoothed data with a moving average of 5 days. Data courtesy of Dr Collins and Dr Jeffrey.

Generally, malaria within-host models are formulated to examine the mechanisms that can reproduce one or a few characteristics of malaria therapy infection data [30,34,35,37] illustrated in figure 1, including (i) the remarkably consistent height of the initial peak in parasitaemia around 10^5^ parasites per microlitre (figure 1*a*); (ii) following this initial peak, the almost complete disappearance of parasites from nearly 60% of patients, sometimes for more than 20 days, and subsequent recrudescence (figure 1*b,d*); (iii) the slow log-linear decay in peak densities (figure 1*c*) [41] and (iv) long-scale oscillations in parasitaemia occurring subsequently, obscured by daily fluctuations resulting from the 48 h life cycle of the parasite (figure 1*e*).

In the absence of constraints, the relatively unconstrained exponential parasite growth in the blood, observed during early infection, is associated with the intracellular development of a single parasite into 16–30 progeny over the course of a single replication cycle lasting 48 h [42]. In order to reproduce the complex dynamics observed throughout the entirety of an infection within a mathematical model, however, this intrinsic capacity for rapid growth must be controlled by a combination of resource limitation, via the availability of susceptible red blood cells, and various mechanisms of immunity that either prevent invasion or clear the parasite [10,30,43].

### 2.2. Biological assumptions of a within-host discrete model of blood-stage parasitaemia

We develop a discrete, deterministic within-host mathematical model with parameters chosen stochastically to examine the conditions under which commonly assumed interactions between the host immune response and the parasite actually lead to the infection dynamics described in figure 1. We choose assumptions based on their biological plausibility and widespread use in the modelling literature, and incorporate co-infection to examine the validity of assumptions of many transmission models of malaria. Methods are given in the Detailed Methods section, but in brief, we can separate the assumptions into parasite and host components.

To portray diversity of parasites, we model antigenic variation within the host. As in previous models [12,30,32,33,37,40], we assume that during a single infection, *P. falciparum* parasites systematically alter the proteins they present on the surface of infected red blood cells in a process mediated by *var* genes, which produce the *P. falciparum* erythrocyte membrane protein 1 (*Pf*EMP1) [44–49]. Parasites are assumed to have 60 possible *var* genes, although recent evidence suggests that this may be an underestimate as the number of *var* genes may increase during asexual replication owing to mitotic recombination [50]. While the number of *var* genes for a genotype is fixed, each parasite expresses a single *Pf*EMP1 type on an infected red blood cell at any time [46,51–54], and they switch the gene they are expressing at a variable rate [55,56]. We examine variation in the variant switching network, number of initial variants expressed, and the distribution of variants between cross-reactive (CR) groups. We do not explicitly model other parasite antigens but consider the effect of cumulative exposure to these antigens throughout the infection.

**Figure 2.**
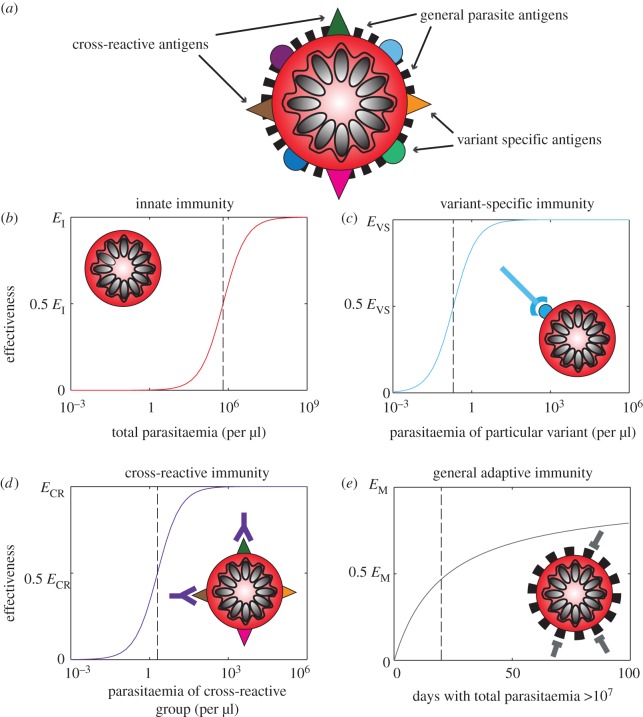
Schematic of the four types of immunity in the model. (*a*) An infected red blood cell is shown schematically with illustrative surface antigens. Black bars are antigens that do not vary. Coloured circles and triangles are antigenically varying antigens. (*b*) Innate immunity reacts equivalently to all parasite-infected red blood cells. (*c*) Variant-specific adaptive immunity reacts to particular subpopulations of parasite-infected red blood cells, which display identical antigens (blue circles). (*d*) Cross-reactive adaptive immunity reacts to particular subpopulations of parasite-infected red blood cells, i.e. those that display similar antigens in CR groups (triangles of various colours). Both the VS response and the CR response grow as the ratio of antigen to antigen-specific cells increases including an initial delay for production of specific immune cells. (*e*) General adaptive immunity builds slowly through the course of infection as the number of exposures to high parasitaemia (>10^7^ parasites) accumulates.

We include four types of immune responses—innate, variant-specific (VS) adaptive, CR adaptive and general adaptive—that respond to various populations of infected red blood cells. Both the innate and general adaptive responses act equivalently upon the entire parasite population, with the innate response responding directly to the total level of parasitaemia while the general adaptive builds with the cumulative burden of antigen experienced throughout the infection. VS responses counter parasites that display identical variant antigens while CR responses act against populations with similar but not necessarily identical variant antigens. We assume that host immune responses generally follow a decelerating growth curve or type II functional response with respect to the amount of antigen available, and incorporate a time-lag for activation in adaptive responses (figure 2). As the antigen levels shift exponentially with the growing parasite population, these immune responses appear sigmoidal with a steep switch, from minimal to high response, with small changes in the parasite population, which we will refer to as a threshold. We consider the threshold, and level of activation and decay of immune responses to be inherent characteristics of a host but vary these among individuals, representing heterogeneity of host immune responses. We conduct extensive sensitivity analyses of all parameters (table 1) simultaneously through a Latin hypercube sampling scheme [62].

**Table 1.** Parameters ranges of discrete within-host mathematical model.

symbol	description	value	range
*γ*_*i*_	intrinsic growth rate of variant *i*	16	[0,32] [57]
*ω*_*i*_	percentage switching to a new variant from variant *i*	2	[0,20] [58]
*β*_*ji*_	switching probability from variant *j* to variant *i*	1/60	[0,1] [59]
***—***	number of starting variants	5	[1,60] [60]
***—***	number of cross-reactive groups	5	[1,2,5,10,20]
*E*_I_	maximum efficacy of innate immunity	0.95	[0.7,1]
*C*_I_	half-maximal activation of innate immunity	10^9.5^	[10^7^,10^11^]^a^
*E*_VS_	maximum efficacy of variant-specific immunity	0.8	[0.5,1]
*ψ*_VS_	half-maximal activation of variant-specific immunity	10^6^	[10^5^,10^10^]^a^
*E*_CR_	maximum efficacy of cross-reactive immunity	0.8	[0.5,1]
*ψ*_CR_	half-maximal activation of cross-reactive immunity	10^9^	[10^5^,10^10^]^a^
*E*_M_	maximum efficacy of general adaptive immunity	0.95	[0.5,1]
*C*_M_	half-maximal activation of general adaptive immunity	20	[10,100]
*K*(0)	restriction of total number of red blood cells	10^13^	—
*K*_Imm_	restriction of total number of immune cells	10^14^	—
*μ*_VS_	maximum decay rate of variant-specific immune cells	0	[0,1]
*μ*_CR_	maximum decay rate of cross-reactive immune cells	0	[0,1]
*π*_VS_	maximum growth rate of variant-specific immune cells	8 [61]	—
*π*_CR_	maximum growth rate of cross-reactive immune cells	8 [61]	—
*τ*	delay of adaptive immune response activation (days)	10 [61]	—

^a^Indicates the interval was sampled uniformly on a logarithmic scale.

### 2.3. Length of modelled malaria infection varies considerably, even among similar parasites in identical hosts

**Figure 3.**
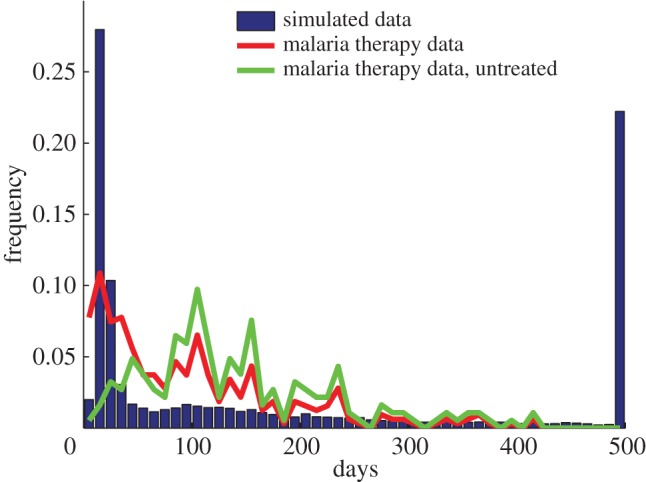
Length of infections of malaria therapy data and simulated data. Histograms of the proportion of replicates with a given length of infection for simulated data (blue bars), malaria therapy data including all patients (red line) and malaria therapy data including only untreated patients (green line). The simulated data exhibits three peaks: the first from acute infection, peaking near 20 days; the second from cleared chronic infections, peaking near 100 days; the final peak owing to numerical termination of the simulation at 500 days. The malaria therapy data (red line) shows an initial peak near 20 days, primarily of individuals whose parasitaemia spiked and required treatment. Another peak occurs near 100 days, with many chronic infections of varying length. The untreated malaria therapy data (green line) does not have the early peak and instead peaks near 100 days with a wide range of infection lengths.

**Figure 4.**
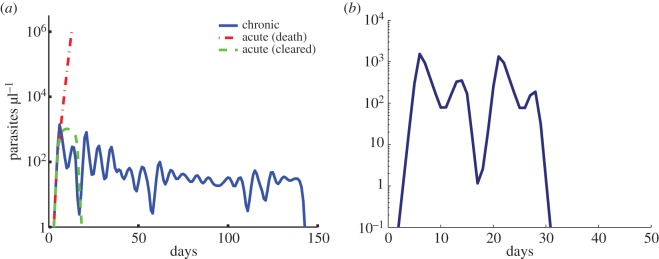
Sample simulated trajectories of parasite dynamics within individual hosts. (*a*) Two types of acute infections occur: one (red dotted) where the host is killed and the other (green dashed) where the immune response clears the parasite quickly. Infections can also extend to weeks or months (blue) before they are cleared. (*b*) In the absence of the general adaptive immune response, the peaks in parasitaemia do not decay through the infection and clearance occurs abruptly.

**Figure 5.**
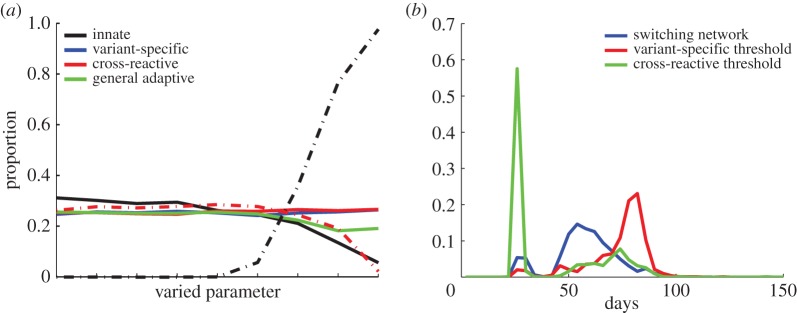
Sensitivity analysis of parameters. (*a*) Histograms of the proportion of simulations that last longer than 50 days. All parameters are varied simultaneously under a Latin hypercube sampling scheme and are binned here by individual parameters to show the impact of immune thresholds and efficacy on chronicity. The innate immune threshold is shown with a black solid line and is varied on a linear scale [10,100]. The variant-specific immune threshold is shown with a blue solid line and is varied on a log scale [10^5^,10^10^]. The cross-reactive immune threshold is shown with a red solid line and is varied on a log scale [10^5^,10^10^]. The general adaptive immune threshold is shown with a green solid line and is varied on a log scale [10^7^,10^12^]. The innate efficacy is shown with a black dashed line and is varied on a linear scale [0.5,1]. The VS efficacy is shown with a blue dashed line and is varied on a linear scale [0.5,1]. The CR efficacy is shown with a red dashed line and is varied on a linear scale [0.5,1]. The general adaptive efficacy is shown with a green dashed line and is varied on a linear scale [0.5,1]. (*b*) Histograms of varying infection lengths of nearly identical parasites with differing switching networks in identical hosts (blue), identical parasites in hosts identical except for different VS thresholds (red), or identical parasites in hosts identical except for different CR thresholds (green). In the nearly identical parasites, the switching networks have unique individual switching connections between variants but are fully connected with biased switching towards a small number of variants. Parameters are standard values from table 1 unless otherwise specified.

Our model produces infections that vary enormously in length, from 20 to over 500 days under reasonable biological assumptions for parameters (figures 3 and 4*a*), and the simulated infection lengths often show a bimodal distribution (figure 3). Here, infections are either short owing to host death or clearance following a single large peak in parasitaemia, or persist with a rebound following the initial drop in parasitaemia. Among this second group of infections, a further distinction can be made between simulated infections that display decaying peaks over long timescales that are eventually cleared by the adaptive immune response, and some lasting much longer (the maximum simulated time) owing to the control and persistence of parasites at low density by the combination of adaptive immune responses. Over a wide range of parameters approximately 30% of simulations last longer than 50 days with little variation in the fraction of simulations producing such chronic infections when each parameter is considered separately (figure 5*a*), with the exception of the efficacy of the innate immune response, which favours chronicity at larger values.

Interestingly, we observe large heterogeneity in length of infection even among similar parameter sets (figure 5*b*). Parasites with nearly identical characteristics—the same number of CR groups, switching rates and connections drawn from an identical distribution, and the same number of starting variants—can produce very different infection lengths in identical hosts. For example in figure 5*b*, simulations where parasites start with five variants present and have their variants split into five equally sized CR groups but differ only in their variant switching network (blue line) lead to infections from 20 to nearly 100 days (figure 5*b*). This results from a complicated interplay between the adaptive immune response and proliferating parasite variant populations. Models incorporating these aspects of infection usually examine only a small range of these parameters, which leads to the incorrect assumption that chronicity is a robust outcome of infection [32,37].

### 2.4. Disappearance of parasitaemia early in a chronic infection cannot be explained by immune responses

A consistent feature of the malaria therapy data is a plummet in parasitaemia following the initial peak (figure 1*b,d*), frequently falling to levels below microscopic detection for several days to several weeks before re-emergence [41]. Although many models are able to reproduce a drop in parasitaemia, which is often caused by a strong antigen-specific adaptive immune response, parasites either are cleared from circulation or remain above microscopically detectable levels prior to re-emerging. Historically, observational studies of malaria therapy data ascribed the resurgence of parasitaemia to antigenic variation [35,63]. Models have been able to reproduce recurrent peaks of parasitaemia via host immunity and resource restriction of viable red blood cells, but not with a drop of parasitaemia below microscopic detectability as seen in the malaria therapy data [10,11,43,64]. Our model also struggles to reproduce submicroscopic parasitaemia prior to re-emergence, indicating the incomplete understanding of the basic mechanisms of immunity underlying this dynamic.

### 2.5. Antigenic variation is not a robust driver of chronicity

It has often been assumed that the long duration of infections observed in the malaria therapy data are mediated by antigenic variation of the approximately 60 *Pf*EMP1 surface antigens encoded by the *var* gene family. Various studies have evaluated aspects of antigenic variation including switch rate, switching order and clearance rates [46,51,54,58,60,65–68], but modelled antigenic variation also depends on the number of variants expressed at the outset of the infection, with chronicity being promoted by fewer starting variants [32]. Recent data from *in vitro* experiments suggest that nearly all variants may be expressed at the outset of an infection [69–71], presenting a significant challenge to our conceptual understanding of the orchestration of antigenic variation [60]. Choosing parameters based on experimental evidence pointing to switching rates between 0.5% and 20% [58] and a highly connected switching pattern with variants able to switch to almost all variants [59], all variants appear within the first 10 generations of a modelled infection. Even with conservative choices for parameters associated with antigenic variation—switch rates where less than 2% of parasites switch per replication cycle, switching networks constrained such that variants are able to switch to no more than two variant types, and less than five starting variants—we find that new variants appear rapidly such that all 60 *Pf*EMP1 variants are expressed before the end of the infection. However, it may take significantly longer for variants to elicit immune responses. Our model therefore implies that even with the most conservative switching estimates, parasite-driven mechanisms of antigenic variation alone are not sufficient to robustly produce the long-lived chronic infections as observed in the malaria therapy data.

### 2.6. Theoretical models of cross-reactive immune responses with decay lead to chronic but unrealistic infection dynamics

Recker *et al.* [39] demonstrated that CR immune responses are capable of lengthening an infection without any antigenic changes by the parasite, by limiting parasite variants expressing CR proteins. However, a key assumption of this model was the rapid decay of CR responses relative to VS response. Johnson *et al.* [33] also recovered chronicity in the presence of CR immune responses but without decay of these responses when immune cells become less effective as the infection proceeds, owing to saturated killing or immune exhaustion. Whenever CR responses are present, it is possible that some variants are suppressed and do not elicit a strong enough VS immune response for clearance. As cross immunity wanes, whether owing to decay [37,39] or exhaustion [33], previously suppressed variants arise and proliferate in the population. Because these models are designed to examine only the mechanism of chronicity, they fail to reproduce realistic infection dynamics with later peaks routinely reaching the same height as the initial peak, a phenomenon not observed in natural infections. When lacking a general adaptive immune response in our model, similar to the assumptions in these models [33,39], we find that peak height does not decay over time (figure 4*b*). The theoretical description of standard innate and adaptive immune responses is therefore lacking a key component that can result in the characteristic decay of parasite density at peaks found in natural infections (1).

### 2.7. Characteristic decay of parasite density is difficult to describe theoretically

The failure to produce the nearly log-linear decay of peak density from an initial peak (figure 1*c*), which is one of the hallmarks of malaria infections [41], represents a significant challenge to modellers. In the absence of drugs, this decay is consistent across patients until late in an infection when parasite counts become unreliable owing to stochastic fluctuation around the limit of detection. In our model, we can achieve this gradual decay in parasitaemia, similar to previous models, by applying an adaptive immune response that is cross-reactive among all variants and slowly grows over the course of infection [13,30] (figure 2*e*). We do not assume any decay in this response, and it grows whenever there is parasite density above a defined threshold. Although recent experimental evidence has identified cross-reactive antibodies, there is no evidence they lack the ability to decay or that they increase only when parasitaemia is above a threshold [72–74]. Although we, like others, can achieve a qualitatively similar decay in parasitaemia using this function [12,13,30], we propose that this widely used immune function lacks robust biological evidence, and represents a major challenge in our understanding of malaria infection dynamics.

### 2.8. Co-infection alters infection length

Dynamics of parasite populations in individual hosts result in very heterogeneous lengths of infection, which will significantly impact the length of time individuals are infectious to the mosquito vector. Particularly, in the areas of high endemicity, the length of infection will be further confounded by the presence of multiple infections in a single host. Few transmission models include co-infection and those that do make implicit assumptions about the impact of co-infection on infection length and competition and, furthermore, rarely include heterogeneity in these effects. Here, we use our model framework to explore the implications of our assumptions about immunity and antigenic variation for co-infecting genotypes in a setting where chronicity is common, occurring 80% of the time, when only a single genotype is present.

**Figure 6.**
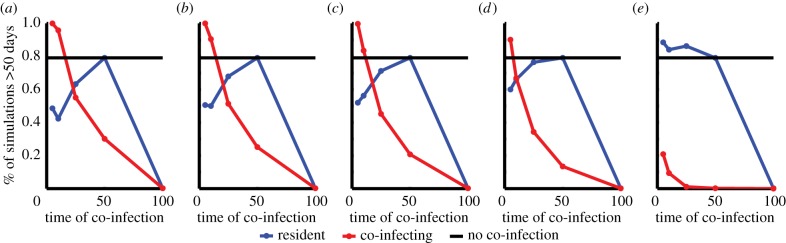
Timing and similarity of co-infection affects success of both the resident and co-infecting strain. The percent of simulations that last longer than 50 days when varying similarity of co-infecting genotypes among VS antigens—no overlap (*a*), 25% overlap (*b*), 50% overlap (*c*), 75% overlap (*d*) and 100% overlap (*e*). Overlap refers to the percentage of VS antigens that are identical between genotypes. The co-infecting strain (red line) is favoured over the resident strain (blue line) at lower similarity and early during an infection. Parameters used are standard values in table 1 with only variation in the switching connections and switching rates. Without co-infection nearly 80% of genotypes lead to infections of over 50 days (black line).

We find that infections comprising more than one strain, even if entering the host at similar times, often lead to enhanced infection length (figure 6). In general, this occurs because of the increased variability in antigens presented to the immune system, which requires more absolute immune pressure for clearance. Parasite genotypes that co-infect during periods of the infection where non-specific immune responses are high—typically early or late in an infection—are at a significant disadvantage (figure 6). Early co-infection accelerates activation of the innate immune response, limiting the total parasitaemia of both genotypes at an earlier time point but at a similar peak parasitaemia level. Late in an infection when there exists a multitude of adaptive immune responses to conserved parasite proteins, the co-infecting parasite is unable to grow appreciably and has a short infection even if the antigenically varying proteins it expresses are distinct.

Interestingly, we find that parasites co-infecting after the initial innate immune response has waned but before the general adaptive response dominates have an advantage over the resident parasite, lengthening the overall infection (figure 6). This implies that there may be an optimal window for co-infection that will determine the outcome of competitive interactions between genotypes. Co-infecting parasites expressing similar antigens to the resident strain are subject to shared adaptive immune responses, and are often suppressed as a result, whereas antigenically distinct co-infecting parasites grow rapidly and either overtake the resident population or cause a strong immune response, altering the length of infection for both parasites (figure 6). The interplay between the growth of the parasite genotypes and the impact of the host immune responses is often subtle and results in both longer and shorter infections, and therefore, simple assumptions about the impact of co-infection on infection length are not supported theoretically.

## 3. Conclusion

Chronic infections are often very difficult to generate robustly in mathematical models under biological conditions and assuming variation in host and parasite traits. Our results demonstrate a curious lack of stability of the infection length to small perturbations of parameter values. Further, co-infection of competing genotypes can profoundly impact the length of infection and transmission potential of both genotypes in a non-intuitive manner, given standard assumptions. Our study therefore suggests that either (i) current models capture the basic mechanisms driving host–parasite interactions and occasional, stochastic chronicity is the norm for malaria infections, or (ii) we are currently missing an important mechanism that reliably generates chronic infections. We propose that the latter is more likely; in particular, we highlight the incomplete understanding of the interactions between the host immune response and the parasite. This distinction is important, because without an accurate understanding of the interplay between the host immune response and the parasite, we will not be able to reliably predict outcomes of intervention strategies.

## 4. Detailed methods

We developed a discrete-time deterministic model of the blood-stage parasite dynamics of malaria infections with stochastically varying parameters. The model is motivated by previous work by Recker *et al.* [37]. Equations and parameters are described in detail below with values for all parameters found in table 1. Parameters are fixed at the beginning of a simulation and remain constant throughout, but are sampled from within the ranges indicated in table 1. Sampling of parameters was done stochastically using a Latin hypercube sampling [62] with the intervals listed in table 1 split into 10 uniform subintervals. The subdivision used a linear scale except for the half-maximal activation of immune responses which was done on a log scale, indicated with a superscript a in table 1. Results presented here vary all or some of the parameters listed, as indicated in the figure legends, and include a minimum of 100 000 replicates. Simulations were completed in Matlab 2013a. Code is available from the authors on request.

Parasites are grouped by their specific antigenic variant, of which each genotype harbours 60 unique antigenic variants. The overall number of parasites of the *i*th antigenic variant is
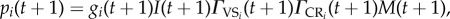
where *g_i_* is the generational growth of variant *i*, *I* is the effectiveness of the innate immune response, *Γ*_VS_ is the effectiveness of the VS adaptive immune response, *Γ*_CR_ is the effectiveness of the CR adaptive immune response, and *M* is the effectiveness of the general adaptive immune response. Each component of this equation is described in detail below. The time step is 48 h, equivalent to one asexual parasite generation in the blood stage. Each simulation begins with 40 000 parasites, equally comprised of the predetermined number of starting variants, which are chosen randomly from the 60 variant types. When more than one parasite genotype is present, each genotype begins with 40 000 parasites. The 60 variants of each genotype may or may not be the same. For any identical variants between genotypes, the adaptive immune response against those variants acts equivalently towards both genotypes.

In the absence of an immune response, the growth of a parasite variant depends on the intrinsic growth rate of the variant and the inherent switching between variants. The generational growth of the *i*th variant, *g_i_*, is determined by
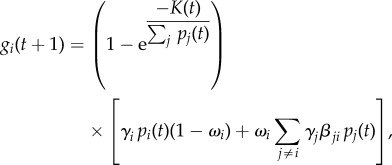
where the first term on the right-hand side restricts the growth of parasites when red blood cells are limited, and the second term determines the switching and growth of parasite variants. When the number of available red blood cells, *K*(*t*), falls below one-third of the original value, the host is categorized as dead. During each time step, *K*(*t*) is reduced by the number of newly infected red blood cells and grows by *K*_Imm_/120. The growth rate, *γ*_*i*_, of each variant is chosen from a normal distribution with mean 16 and variance 8. If the growth rate falls below 1, it is set to 1, and if it is above 32, it is set to 32. The rate of variant switching, *ω*_*i*_, is chosen uniformly on the interval zero to two. The probability of switching from variant *j* to variant *i*, *β*_*ji*_, allows all variants to switch to all other variants, such that each entry is positive except along the diagonal. Each row of *β* gives the probabilities of switching from variant *j* to any other variant and thus sums to one. The same does not hold for the columns as the values of *β*_*ji*_ are biased to favour some variants with higher probability as described by Nobel *et al.* [59]. The results do not require a switching structure such that all variants can switch to all other variants. In fact, the results are robust to the structure *β*_*ji*_, except when the switching behaviour is strictly ordered such that each variant only switches to a single other variant, which implies the matrix *β* is sparse. The values for *ω*_*i*_ and *β*_*ji*_ are varied for each simulation unless otherwise noted. Variants in each parasite are placed in CR groups mimicking the overlapping immunity experienced by similar antigens. The variants are placed into the predetermined number of CR groups randomly.

The immune response to parasites is multi-faceted, beginning immediately with an innate response that acts equally upon all variants. The effectiveness of the innate immune response, *I*, is dependent on the total parasite population
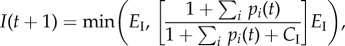
where *E*_I_ is the maximal efficacy of the innate response and *C*_I_ is the level at which the innate immune response is half-maximal. Unlike the innate immune response, which occurs immediately, the VS and CR components of the immune response require a delay of *τ* days for immune cells to be created. The VS immune response acts uniquely upon each parasite variant, similar to the individual immune responses to each of the various *Pf*EMP1 surface proteins [48]. The CR immune response acts similarly towards groups of variants rather than single variants but is generally less effective at killing parasites compared to the VS response. The dynamics of the VS and CR adaptive immune cells are governed by
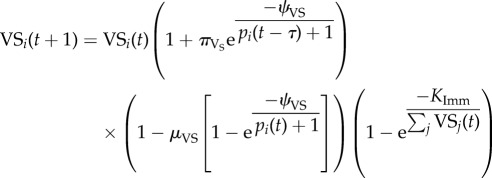
and
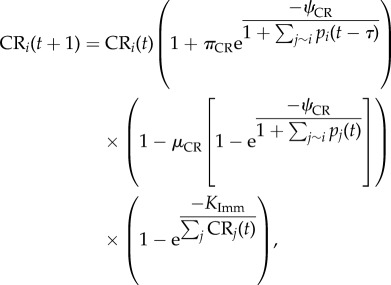
where the first term is the pre-existing cells of the response, the second term is the expansion of the immune cell population when antigen is present in large quantities, the third term is the decay of the immune cells when antigen is only present in small quantities or not at all, and the final term limits the total number of immune cells. For CR responses, *j* ∼ *i* refers to all variants that are from the same CR group. There is a minimum of 100 immune cells responsive towards each variant present at every time point even prior to *t* = *τ*. The growth (*π*_VS_, *π*_CR_) and decay (*μ*_VS_, *μ*_CR_) rates of immune cells determine the immune cell population and is mediated by the level when the response is half-maximally activated (*ψ*_VS_, *ψ*_CR_).

The effectiveness of the variant-specific (*Γ*_VS_) and cross-reactive (*Γ*_CR_) immune responses depends upon the relative amount of parasite variants and specific immune cells
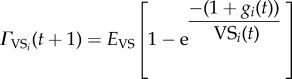
and
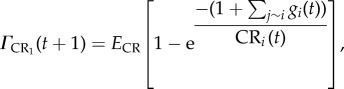
and are capped by the maximum efficacy (*E*_VS_, *E*_CR_) of each response.

The final component of the immune response is the general adaptive immune response, which acts identically upon all variants but builds slowly over the course of the immune response through the repeated exposure to antigens. The effectiveness of the general adaptive immune response, *M*, depends on the cumulative numbers of days during the infection when the parasite population is above 10^7^
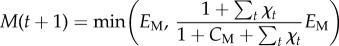
and
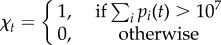
where *χ*_*t*_ is an indicator function, *E*_M_ is the maximum effectiveness of the general adaptive response, and *C*_M_ determines the time at which the response is half-maximal.
